# Tuberculosis in Humans and Cattle in Jigawa State, Nigeria: Risk Factors Analysis

**DOI:** 10.1155/2012/865924

**Published:** 2012-07-12

**Authors:** S. Ibrahim, S. I. B. Cadmus, J. U. Umoh, I. Ajogi, U. M. Farouk, U. B. Abubakar, A. C. Kudi

**Affiliations:** ^1^Department of Veterinary Surgery and Medicine, Ahmadu Bello University, P.O. Box 720, Zaria, Nigeria; ^2^Department of Veterinary Public Health and Preventive Medicine, University of Ibadan, P.O. Box 720, Ibadan, Nigeria; ^3^Department of Veterinary Public Health and Preventive Medicine, Ahmadu Bello University, P.O. Box 720, Zaria, Nigeria; ^4^Animal Reproduction, Unit Jigawa Research Institute, 2646 Kazaure, Nigeria

## Abstract

A cross-sectional study was conducted from September 2008 to March 2009 to identify risk factors for BTB in cattle and humans in Jigawa State, Nigeria. A total of 855 cattle belonging to 17 households were subjected to comparative intradermal tuberculin test (CITT) while interviewer-administered questionnaire was used to obtains information on the risk factors. Twenty-two (22) respondent (5%) amongst the families sampled had TB or clinical signs suggestive of TB, while 9 (2%) had reactor cattle in their herds; However, no statistically significant association (*P* ≥ 0.05) was observed between reactor cattle and human TB cases in the households. The habit of milk and meat consumption was found to be affected by occupation and location of the household residence. None of these risk factors (food consumption, living with livestock in the same house, and presence of BTB-positive cattle) were found to be statistically significant.

## 1. Introduction

Bovine tuberculosis (bTB) is an infectious disease caused by *Mycobacterium bovis*, a member of the *Mycobacterium tuberculosis* complex (MTC), which comprises the closely related *M*. *tuberculosis*, the major causative agent of human tuberculosis (TB) [1]. Worldwide TB caused about 2 million deaths and about 9 million new cases had been reported annually, with sub-Saharan Africa having the highest annual risk of infection with TB, probably catalyzed by the HIV/AIDS pandemic [2]. Globally, *M*. *bovis* accounts for 3.1 percent of all human TB cases [3]. However, the extent of *M*. *bovis* involvement in the global TB burden in Africa is still largely unknown. This can be partly explained by the fact that in humans, TB due to *M*. *bovis* is indistinguishable from that due to *M*. *tuberculosis* in terms of clinical signs, and radiological and pathological features [4]. In addition, most laboratories in sub-Saharan Africa do not have the capability to differentiate *M*. *bovis* from *M*. *tuberculosis* [5]. Although cattle are considered to be the main hosts of *M*. *bovis*, the disease has been reported in many other species, including humans, other domesticated animals, and wildlife [6].

Indeed, little information on risk factors of disease transmission to cattle, between cattle and from cattle to humans, is available from an African context. Most information are extrapolated from experiences in developed countries. For Africa, the most comprehensive studies done so far have been in Tanzania [7–9] and Uganda [10]. However, despite the lack of information, it is generally accepted that besides causing major economic losses and also poses a serious zoonotic threat in Africa [5].

Nigeria has a population of over 130 million people and ranked 4th among the world's 22 countries with high TB burden, [11]. Among African countries, Nigeria has the highest estimated number of new cases with nearly 368000 new cases annually [12]. In countries where bovine TB is uncontrolled, most human cases occur in young persons and results from drinking or handling contaminated milk [3]. Little is known of the frequency with which *M*. *bovis* causes nonpulmonary TB in developing world due to the limited laboratory facilities for culture and typing of tubercle bacilli [3]. In Nigeria, the only method used in the diagnosis of suspected TB case is by direct smear microscopy which does not permit the differentiation of *M*. *tuberculosis* complex. This could partly explain the relatively low notification rate of the diseases caused by *M*. *bovis*.

In this paper, we attempt to mainly assess possible risk factors for BTB in cattle and humans in Jigawa State, Nigeria.

## 2. Materials and Methods

### 2.1. Designs

A cross-sectional survey was conducted from June 2003 to July 2006 in twelve local government areas of Jigawa State, Northwest part of Nigeria, in conjunction with parallel study on tuberculosis testing on cattle. Four hundred and thirty-five households were interviewed about knowledge, attitudes, and practices with regards to tuberculosis control. 

### 2.2. Study Area

Jigawa State lies between longitude 11° to 130 North and latitude 80° to 100 East, with a landmass of 22, 140 km^2^ [13]. The state has 27 local government areas with about 350,000 of the rural households keeping livestock [13].

The human population of the district is estimated at 4.3 million (NPC, 2006). National Population Commission (2007). Nigerian National Census Figures. The total number of households in the district was 350,000 and each household has an average of 5 individuals. The cattle population in the state comprises mainly of white Fulani (Bunaji) and the Rahaji breeds also known as the Red Bororo. The common husbandry system practice in the state is the extensive management system, although, nowadays the herd owners are becoming settled pastoralists.

### 2.3. Study Subjects and Procedures

A list of villages for each local government was obtained at the local government headquarters using a random table, 12 villages in the three emirate councils were selected.

In each village, two samples were obtained, one from the population that keep livestock and are also crop farmers and another from the group that are livestock keepers. The populations that keep livestock as well as crop farming are designated as agropastoralist population for the purpose of this study and were selected based on consenting individuals (the willingness of the individuals to participate).

The pastoralist group consisted of livestock keepers whose cattle were screened for tuberculosis. Home visits were made a day after the tuberculin testing. For all the two groups, verbal consent was obtained to participate in the interview. The family then selected a member, usually the head of the family or older member, depending on their availability for the interview.

### 2.4. Tuberculin Testing of Cattle

The comparative intradermal tuberculin test was conducted in all cattle using both avian and bovine purified protein derivatives (PPD) supplied by the Veterinary Laboratories Agency, Weybridge, UK. Intradermal injections of 0.1 mL (2500 IU/ mL) bovine PPD and 0.1 mL (2500 IU/ mL) avian PPD were administered in two shaved areas, 12 cm apart from each other in the middle of the neck, after having measured and recorded skin thickness with a vernier caliper. Skin thickness was measured again at both injection sites after 72 hrs. The reaction at each site was derived by measuring the difference of the skin thickness before and 72 hrs after the injection. An animal was considered positive if the bovine minus the avian reaction was greater than 4 mm. 

### 2.5. Questionnaire

Field workers who were trained as extension agents and who are local government employees were given the questionnaires to administer to the various groups.

The status of milk for consumption was recorded as boiled if both fresh and soured milk types were boiled, and not boiled if one or both types were not boiled. The history of TB in the family was based exclusively on previous hospital diagnosis. Name, sex, and age of patients were recorded as obtained on their hospital records. The interviews were held with people who were ready to give information. Interviews were conducted on the same day their cattle were being tested for BTB.

### 2.6. Data Analysis

The data obtained was entered into a personal computer. Analysis was done using *Epi info 2002 (Revision 2) *statistical software. Chi-square and Fishers exact test were used to analyze various factors was applicable.

## 3. Results

### 3.1. Demographic Characteristics

The study comprised of 278 (63.91%) males and 157 (36.10) females (*n* = 435) with no significant variation between the two groups (*P* < 0.05) (Table 2).

### 3.2. Food

The proportion of households drinking milk were 82% (*n* = 357) and 93% (*n* = 405) in the agropastoralist and pastoralist groups, respectively (Table 3). Milk was boiled by 67.82% (*n* = 295) and the variation between the two groups was not significant.

Under cooked meat and meat products were not consumed by the entire respondents in the study areas. Meat and meat products were eaten at least monthly by 58.40% of the respondents (*n* = 254).

### 3.3. Housing

The distribution of types of housing and ventilation is indicated in Table 4. Houses with inadequate ventilation were observed in 66.35% of the agropastoralist, while 86.05% was observed in the pastoralist group and there was significant variation between the two groups (*P* = 0.283). On the average, more than 50% of the households in the study population reported at least one activity considered a risk factor for transmission of zoonotic tuberculosis (Table 1). These activities were mainly related to handling animals and animal products, and include milking, slaughtering, and living in the same house with animals. These activities were more in the pastoralist groups, than the agropastoralist groups (Table 1).

Analysis of risk factors associated with the occurrence of the disease in this area (Table 5) revealed that the reactor rate among the age groups differ significantly (*P* < 0.01), with older cattle affected more than younger cattle and calves. The proportion of indigenous cattle reacting positively was significantly higher (*P* < 0.05) than that of the exotic breeds. More male cattle were affected than females. There was no significant association between pregnancy and reactivity to CIDT, but significantly more lactating cows than nonlactating cows were positive.

## 4. Discussion

This study indicates that most families in the pastoralist group consume fresh or soured milk regularly throughout the year, while the agropastoralist groups boil milk before use. These findings are similar to those of Walshe et al. [14], who reported that about 90% of milk is consumed fresh or soured in sub-Saharan Africa and that *M. bovis* is generally destroyed by boiling or during souring process. However, *M. bovis *has been found to persist for up to 14 days [15]. *M*. *bovis* and *M*. *tuberculosis* have been found in milk samples in Nigeria [16].

Consumption of meat and meat products show some variation between pastoralist and the population as a whole. In all the groups about a quarter of the households have practiced at least one habit considered to pose a risk for transmission of *Mycobacteria* as well as other zoonotic pathogens such as brucellosis. The potential risk of *Mycobacteria* transmission by these means is also reported in other studies [3, 17].

The livestock-related activities reported in this study and especially in pastoralist groups pose a potential risk of *M. bovis* transmission via aerosol or as a consequence of bacterial excretion in faeces, urine, and milk of infected cattle [3]. The situation observed in the sampled areas are favourable for BTB transmission among animals (close contact, poorly ventilated houses, crowded watering and pasture places), very few animals were found to be positive. This indicates a low transmission rate in the investigated livestock.

Relative lack of knowledge among pastoralist as compared to general population group may provide an explanation for low levels of public health awareness, as families of livestock keepers are not able to attend schools due to their nomadic nature compared to permanently settled households.

The proportion of households in which animals were confined in the same house with families is higher in the general population group. It is more likely for families in the agropastoralist group to have fewer livestock and such families tend to confine their animals in their own houses. Inadequate ventilation and confining of livestock in the same house with the family might play a major role in transmission of tuberculosis in the study area. Lack of ventilation could also enhance aerosol transmission of *M. tuberculosis* as well as *M. bovis* from cattle to cattle or from cattle to humans. The study also found that keeping livestock in the same house at night may serve as a risk factor for tuberculosis, suggesting a zoonotic component of transmission [18]. Close proximity in combination with poorly ventilated environment may be a risk factor for occurrence of animals to humans aerosol transmission. The reported human TB in our study comprised all forms of the disease and no differentiation was made between *M. bovis* and *M. tuberculosis*. Farmers consumption habits (raw milk, and products) did not show any statistical significance as reported previously in Ethiopia [19]. We did not find any correlation between PPD, positive cattle, and human case of TB in the household, even though people and cattle often shared the same house. However, statements on the zoonotic potential of BTB require confirmed *M. bovis* cases to address their risk factors specifically. Likewise, having confirmed TB cases in a household was not associated with cattle reactors.

 The observation that male animal had a higher chance of being positive than female animal in the tuberculin tests conducted, may be related to the usage of the male cattle in agriculture. Male cattle are mostly used as oxen and therefore are kept in the herd for long thereby having more chances of being exposed to infection than female cattle. Similarly, female cattle have less frequent contact with other cattle except at grazing and watering point [20].

Although the current study did not classify lactating reactors according to stage of lactation, a significantly higher (*P* < 0.05) reactor rate was found among lactating than non-lactating animals.

Although the current study did not classify lactating reactors according to stage of lactation, a significantly higher (*P* < 0.05) reactor rate was found among lactating than non-lactating animals. The observation that older cattle were found to be significantly more affected by bovine tuberculosis than young cattle showed that older cattle are positive to the tuberculin test mainly owing to increased chances of exposure rather than the slow progression of the disease [7].

## Figures and Tables

**Table 1:** Risk factors for having TB in a family.

Risk factors	Yes	No	% (Yes)
Awareness			
Aware	273	162	62.76
TB in the family	22	0	5.05
Knowledge			
TB symptoms	205	230	47.13
Can TB spread from animals to man?	198	137	45.52
Can TB spread from man to animals?	127	305	
Milk consumption habit			
Boiling	290	145	66.67
Souring	145	290	33.33
Others (fresh)	243	192	55.86
Health seeking behavior			
Seek veterinary assistance	175	260	40.23
Attempt to treat by owners	207	228	47.59
Cull	334	111	76.78
Slaughtering at home	297	138	68.28
Animals in the same House	257	178	59.08

**Table 2:** Demographic distribution of study sites.

Sample	No of respondents	%
Hadejia		
Hadejia	45	10.34
Auyo	30	6.90
K/Hausa	40	9.20
Birniwa	30	6.90
Gumel		
Maigatari	25	5.75
Sule-Tankarkar	15	3.45
Gagarawa	50	11.50
Gumel	55	12.63
Kazaure		
Kazaure	60	13.80
Yankwashi	30	6.90
Roni	30	6.90
Gwiwa	25	5.75
Occupation		
Cattle keeping only	46	63.91
Farmers and cattle keepers	119	36.10
Sex		
Male	278	63.91
Female	157	36.10

**Table 3:** The percentage of risk practices of eating/drinking of animal and animal products.

Practices	Agro-pastoralist		Pastoralist	
Milk				

Drinking behavior	Yes	No	Yes	No
Fresh	98	337	344	91
Soured	124	311	130	305
Boil	140	295	155	280
Others (boiling + souring)	234	140	150	233

Frequency	Everyday	Weekly	Monthly	

During wet season	227	305	95	
During dry season	64	107	159	

Meat or meat products				

Eating habit	Yes	No	Yes	No
Cooked meat/meat product	405	0	425	0
Eating wild animal meat (Bush meat)	315	106	258	173

^∗^Some totals do not add up to 435 owing to missing information or nonapplicability.

**Table 4:** Risk behavior and practices: housing.

Factors		Agro-pastoralist			Pastoralist	
	Yes	No	*n*	Yes	No	*n*
Mud housing	95 (25.6)	276 (74.40)	371	29 (7.57)	354 (92.43)	383
Leaves/grasses	120 (31.83)	257 (68.17)	377	243 (59.27)	167 (40.73)	410
Ventilation	140 (33.65)	276 (66.35)	416	42 (13.95)	259 (86.05)	301

^∗^Totals do not add up to four thirty-five (435) owing to missing information or nonapplicability.

^∗^Percentages are in brackets.

**Table 5:** Risk factors affecting the reactivity of cattle to tuberculin test in Jigawa State.

Variable	No. of Reactors (%)	*P* value
Age		
Calves <18 month (*n* = 150)	1 (0.67)	0.015
Cattle 1.5–3 yrs (*n* = 343)	0 (0.87)	
Cattle >3 yrs (*n* = 427)	9 (1.4)	
Sex		
Female (*n* = 639)	3 (0.46)	
Male (*n* = 283)	7 (2.4)	0.012
Breed		
Exotic (*n* = 72)	0 (0)	
Local (850)	10 (1.7)	0.442
Pregnancy		
Pregnant (*n* = 153)	0 (0.65)	
Nonpregnant (*n* = 486)	10 (0.41)	0.039
Lactation		
Lactating (*n* = 135)	8 (1.48)	
Nonlactating (*n* = 1)	2 (0.20)	0.000
